# HRTEM low dose: the unfold of the morphed graphene, from amorphous carbon to morphed graphenes

**DOI:** 10.1186/s40679-016-0024-z

**Published:** 2016-08-22

**Authors:** H. A. Calderon, A. Okonkwo, I. Estrada-Guel, V. G. Hadjiev, F. Alvarez-Ramírez, F. C. Robles Hernández

**Affiliations:** 1 Departamento de Física, ESFM-IPN, Ed. 9 Instituto Politécnico Nacional UPALM, 07738 Mexico D.F., Mexico; 2 Department of Mechanical Engineering Technology, University of Houston, Houston, TX 77204-4020 USA; 3 Centro de Investigación en Materiales Avanzados, CIMAV, Miguel de Cervantes 120, 31109 Chihuahua, Chih. Mexico; 4 Texas Center for Superconductivity and Department of Mechanical Engineering, University of Houston, Houston, TX 77204 USA; 5 Instituto Mexicano del Petróleo, Eje Central Lázaro Cárdenas Norte 152, Mexico, DF 07730 Mexico; 6 Center for Advanced Materials, University of Houston, Houston, TX 77204 USA

## Abstract

We present experimental evidence under low-dose conditions transmission electron microscopy for the unfolding of the evolving changes in carbon soot during mechanical milling. The milled soot shows evolving changes as a function of the milling severity or time. Those changes are responsible for the transformation from amorphous carbon to graphenes, graphitic carbon, and highly ordered structures such as morphed graphenes, namely Rh6 and Rh6-II. The morphed graphenes are corrugated layers of carbon with cross-linked covalently nature and sp^2^- or sp^3^-type allotropes. Electron microscopy and numerical simulations are excellent complementary tools to identify those phases. Furthermore, the TEAM 05 microscope is an outstanding tool to resolve the microstructure and prevent any damage to the sample. Other characterization techniques such as XRD, Raman, and XPS fade to convey a true identification of those phases because the samples are usually blends or mixes of the mentioned phases.

## Background

Research in the discovery of carbon nanostructures (CNs) have resulted in two nobel prices: one for the fullerene [1, 2] and the second one for the graphene [3, 4]. These and other CNs are carbon allotropes with nanoscale dimensions (e.g., nanotube, nano onions, etc. [5, 6]). Graphene and the nanotubes are the most interesting when compared to other CNs due to its potential in a wide variety of applications that are derived by its physical properties [7–9]. Thus far, the reported CNs are sp2 bonded, except for diamond that is sp3 [10, 11]. CNs are complex in nature and require sophisticated/dedicated manufacturing infrastructure with rather unfortunately low yields (mg/h) [3, 8, 12–16]. These technological limitations have delayed the true industrialization and commercialization of CNs. Most CNs are relatively good conductors (band gap = 0 eV) except for graphene and diamond that may be considered the best conductors (thermal and electrical) ever discovered [3, 4, 17–19]. Some CNs have shown narrow band gaps in doped (<0.53 eV) [20–23] or in zigzag nanotubes [24, 25]. One of the newly identified phases (Rh6) is a natural semiconductor that makes it unique and worth researching it. We envision that this phase has great potential for electronic and optical applications.

Identification of carbon allotropes is quite complicated due to the large variety of carbon phases and the pronounced effects of even subtle morphology changes as in the twisted layer graphene [26, 27]. The characteristics of the morphed graphenes have been previously predicted numerically [28] and more recently we have proposed their existence [29]. However, there is still a great discrepancy regarding the morphed graphenes (Rh6 and Rh6-II). Here is where the use of electron microscopy becomes important since there should not be any beam-induced damage to the sample so that the genuine crystalline structure and atomic arrangement can be preserved. Additionally until now, the yield of those phases is rather limited and they are normally blends. This combination makes their detection and identification with Raman or other characterization methods (e.g., XRD, XPS, etc.) rather complicated. In the past, those phases have been misidentified as nanodiamonds [30]. Sophisticated ab initio simulations have been carried out to make sure that our findings are correct [29]. We synthesize those structures in a green, friendly, solid-state process [31–33].

### Experimental

Our raw material is the fullerene soot or leftover byproduct from the Krätschmer method [2]. The raw material is a commercial product called fullerene soot which is a consequent leftover material once fullerene is extracted [2]. In this work, batches of 2 g have been milled using a SPEX 8000 M mixer/mill. The milling times have been varied from 0 to 50 h using hardened steel media. Milling times of 50 h or more can contribute to ~2 wt% Fe contamination. Fe is removed using a super magnet and an acid wash (1 h in 6 M HNO_3_) in an ultrasonic bath followed by an ethanol wash. The acid procedure is repeated 5 times.

The characterization includes an X-ray diffraction (XRD), Raman, and high-resolution transmission electron microscopy (HRTEM). The HRTEM is carried in the TEAM 05 tool located at NCEM-LBNL California under low-dose conditions. The dose rate is normally reduced by using the monochromator attached to the microscope, in order to shift the beam without introducing any sort of aberrations, to less than 200 e^−^/Å^2^s for single images and to around 20 e^−^/Å^2^s for images in focal series.

A focal series consists of 40 images taken with an exposure time of 1 s and at different focus settings; between each image, there is a waiting time of approximately 1 s. Thus, the total dose in a focal series reaches approximately 800 e^−^/Å^2^. Such a dose is sufficiently low to prevent damage as a result of beam sample interaction in this investigation. The TEAM 0.5 has been used at 80 kV and aligned to reach a C3 (Cs) aberration coefficient of −0.015 mm with a focus spread of approximately 10 Å. A normal alignment of the TEAM 0.5 has been done by keeping aberrations such as C1, A1, A2, B2, A3, S3, A4, D4, B4, C5, and A5 within the confidence limits of measurement and keeping C3 at the specified value. The conditions have been earlier tested in different materials including graphene [34–36] to image the genuine atomic arrangement of the sample by preventing any damage and keeping the required resolution of the investigated material.

## Results

The CNs phases that we can produce include graphene, graphitic carbon, Rh6, and Rh6-II. The synthesis evolution from amorphous carbon to the morphed graphenes is summarized as follows: amorphous soot (raw), graphene (2–3 layers), graphitic carbon (4–10+ layers), and morphed graphenes (Rh6 and Rh6-II). We are well familiar with amorphous soot and graphene; however, here we will focus on the morphed graphenes that are the product of cold welding during milling [37]. The patent of our process is currently in process [38].

The most intriguing observation is the existence of a morphed structure of Rh6-II type. While the morphing from graphene to Rh6 preserves the sp^2^ bonding, the one to Rh6-II rotates the existing bonds and makes new ones thus changing the bonding character from sp^2^ to sp^3^ (Fig. 2). The development of this transformation sponsors the sp3 bonding that has been monitored using XPS. The content of sp3 in the raw soot is approximately 4.2 wt% and it increases to 18.25 wt% sp^3^ bonding in a sample milled for 20 h of milling. Based on the XPS results, we have an approximate ratio between sp^2^ and sp^3^ of 4.5:1. Some of the sp^2^ belongs to the amorphous carbon and part of the sp^3^ is attributed to dangling bonds; perhaps ~4.2 wt% sp^3^ or slightly more is measured in the raw sample. We attribute this increase in the sp^3^ bonding to the change in nature of the carbon during the morphing from the graphene structures into the non-graphitic or Rh6-II. Yet, there is a critical time (e.g., 20 h) that allows to maximize the presence of sp^3^ and after that it decays.

On a final note, we need to mention that we have the evidence of the first CNs that has a morphing mechanism from sp2 to sp3. A feasibility to detect them using Raman spectroscopy is of paramount importance. Unfortunately, the amounts that we have obtained thus far do not allow us to obtain clear spectra of either phase (e.g., Rh6 or Rh6-II). Therefore, the Raman spectra for the pure phases cannot be resolved due to overlapping of the Raman bands. We have simulated (ab initio using CASTEP) the Raman active modes for Rh6 and Rh6-II structures and compared to preliminary results. Therefore, we have presented experimental evidence for morphed graphene nanostructures in which the carbon–carbon bonding is predominantly sp2 (Rh6) or sp3 (Rh6-II) type.

## Results and discussion

**Fig. 1:**
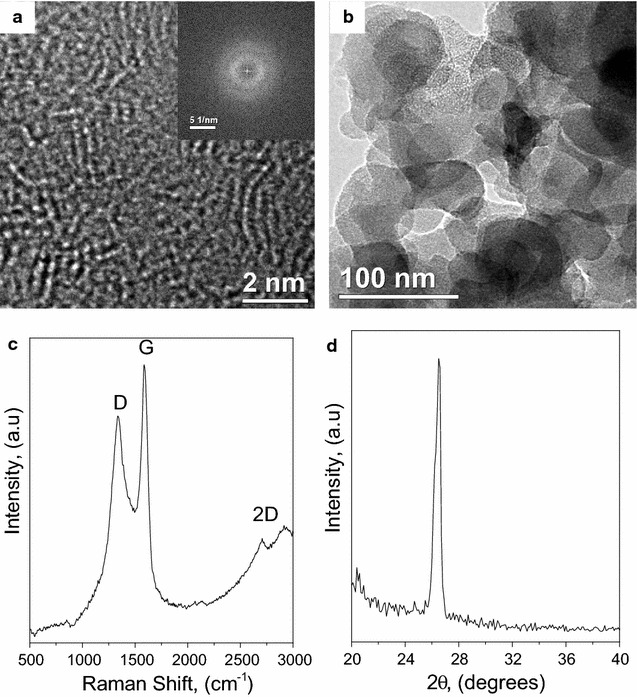
Characterization of the raw carbon soot by means of TEM **a** using HRTEM, **b** bright field, **c** XRD, and **d** Raman Spectroscopy

Figure 1a, b shows HRTEM and bright field images of the raw material (soot) with two main characteristics: the amorphous nature of the soot and the clustering of the soot particles. The XRD spectra of soot show a single reflection at similar d-spacing as that for graphite (002). However, we do not attribute this reflection to the typical ABAB stalking in the “*c*” direction for graphite because our results using the Scherrer method [39, 40] demonstrate that the soot has a grain size of 0.69 nm. This grain size is near that of a single lattice of graphite [30, 41–43]. Therefore, the soot particles are short-range ordered structures and this can be confirmed with the electron microscopy results (Fig. 1a).

Since there are no other observable reflections of graphite in XRD or the FFT-SAEDP (Fig. 1a), we proposed that the soot has limited order in the “*c*” direction and no order in the “*a*” directions. Raman spectroscopy supports the XRD results (Fig. 1d) by the intense D band in the soot. The D band is typical of nanosized or amorphous carbon. The G band suggests a graphitic structure and the weak 2D band indicates that the honeycomb structure is only short range. This combination of features is common in carbon structures with limited order.

Samples were milled from 1 to 50 h and their characterization results were carried out to illustrate the structural changes that occurred in the materials. The main goal is to identify the effect of milling during the synthesis of this new allotrope. In our previous work we reported the existance of those structures [30, 41, 42, 44–46] based on diamond-like polytypes known as 2H and 4H in [47] that are proposed numerically. Here we present the analysis of samples where we show the evidence that these are new carbon nanostructures and should not be confused with neither diamond nor graphitic structures. Instead, we identify them as new nanostructures of carbon that are morphed stacks of graphene and therefore we name them morphed graphene.

**Fig. 2:**
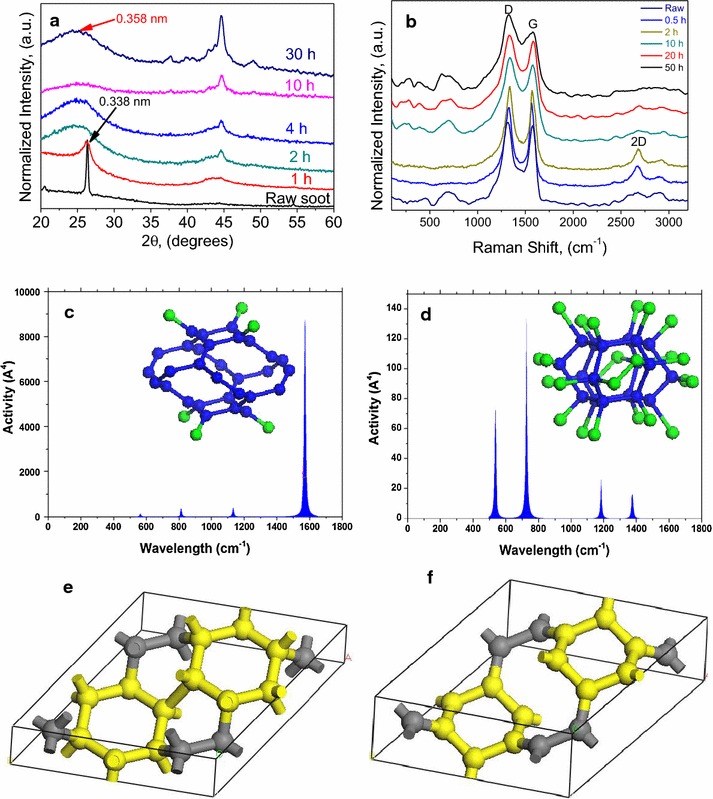
**a** Experimental XRD results for the milled soot from 1 to 30 h, **b** experimental Raman results for the samples milled up to 50 h. (**c** and **d**) ab initio simulated Raman Spectra and respective atomic distributions for Rh6 (**e**) and Rh6-II (**f**) structures

Figure 2a shows the XRD results of the samples milled up to 30 h. In this figure, we can observe from the XRD a series of reflections that do not correspond to single phases; instead, a blend of all the nanostructures are embedded in an amorphous mass. Therefore, only a limited number of reflections are clear and thus the XRD results only allow a limited characterization of the carbon nanostructures. One of the major findings corresponds to the reflection originally observed at 24.85 (2θ) degrees and after a short milling it shifts to 26.3 (2θ) degrees. We cannot interpret this change as an instrumental “shift.” A closer inspection shows that the graphitic reflection widens with short milling times and becomes extinct after 2 h of processing. This phenomenon is followed by the development of the new reflection at 26.3 (2θ) degrees, which is non-graphitic. The respective *d*-*spacings* for the new reflections are between 0.34 and 0.358 nm. These reflections are typical of the (110) planes for the morphed graphenes (Rh6 and Rh6-II) [29]. The Rh6 and Rh6-II structures were first proposed numerically [28] and later on, we demonstrated their existence [29]. In Table 1 are given the experimental results for the *d*-*spacing* for each plane as well as the respective simulated values.

**Table 1:** Identification of phases using the FFT-SAEDP from the images presented in Fig. 6

Particle/FFT-SAEDP	*i*		*ii*		*iii*	
	(nm)	(hkl)	(nm)	(hkl)	(nm)	(hkl)
1	0.309	(101)^a^	0.164	(211)	0.332	(002)
2	0.179	(220)	0.238	(101)	0.162	(004)^a^
3	0.154	(31–1)	0.134	(401)^a^	0.108	(006)^a^
4	0.136	(21–2)	0.217	(300)		
5	0.226	(20–1)				
6	0.115	(330)				

The results correspond to the phases known as (i) Rh6, (ii) Rh6-II, and (iii) graphene

^a^Second-order reflections that are not observed in the ab initio simulations

In Fig. 2b, the Raman spectra for the milled samples for different times are presented. Within short times (0.5–2 h), the 2D band develops, implying that graphene and/or graphitic carbon are still present. Further milling times fade away the well-developed 2D band. The structure of the samples milled for longer times is hard to identify by Raman due to the lack of clarity on the changes, except for features that are present below the 900 cm^−1^. Those features are of interest because graphitic structures are not active in those ranges. In our ab initio simulations for the Rh6 and Rh6-II (Fig. 2c, d) structures, it is observed activity in those regions.

Atomic resolution microscopy and HRTEM are mandatory in this work in order to unfold the characteristics of the as-milled phases. In the following we present the results of low-dose transmission electron microscopy made in the TEAM 05 microscope. The TEAM 05 is selected for this work in principle due to its high stability and development to prevent beam damage by reducing the electron beam dose rate with the use of the integrated monochromator.

**Fig. 3:**
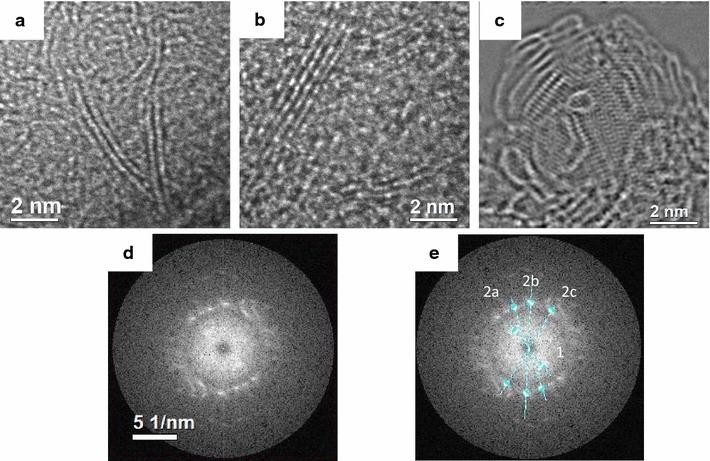
HRTEM images of carbon soot milled for short times, in **a** the presence of double-layer graphene and **b** multi-layer graphenes with up to 5 graphitic layers. The dose rate in use is 197 e^−^/Å^2^s for a pixel size of 0.01883 nm and **c** phase image after exit wave reconstruction procedure. Carbon soot milled for short times, showing the presence of layered graphene parallel and perpendicular to the beam. A focal series of 40 images is used; the dose rate in each case is 55 e^−^/Å^2^s for a pixel size of 0.01883 nm. **d** FFT of phase image in (**c**). **e** Measurement of lattice parameters on FFT

Samples milled for less than 2 h are expected to be graphitic, particularly rich in graphene, and this is demonstrated by the results of XRD and Raman spectroscopy. In Fig. 3, the HRTEM results of the samples milled for 30 min (Fig. 3a) to 2 h (Fig. 3b) are presented. In the shorter times, double layers (Fig. 3a) of graphene with *d-spacing* of 0.33 nm that is comparable to values reported previously are identified [3]. Longer milling times sponsor further cold welding of the graphene layers, transforming it into graphitic structures of 4–5 graphene layers (Fig. 3b). Both structures are quite evident by HRTEM. The background in those samples is amorphous carbon, comparable to that observed in the raw soot. Figure 3c shows a phase image after an exit wave reconstruction procedure with 40 images at different defoci. A blend of phases can be observed in small sections of the sample, with layered graphene being parallel and perpendicular to the beam. Interestingly, little to no evidence of morphed graphene can be found in the Fourier transform (FFT, Fig. 3d) of the image in Fig. 3c. Lattice parameters in the range of 0.197, 0.21 nm and 0.401 nm can be deduced from the FFT.

**Fig. 4:**
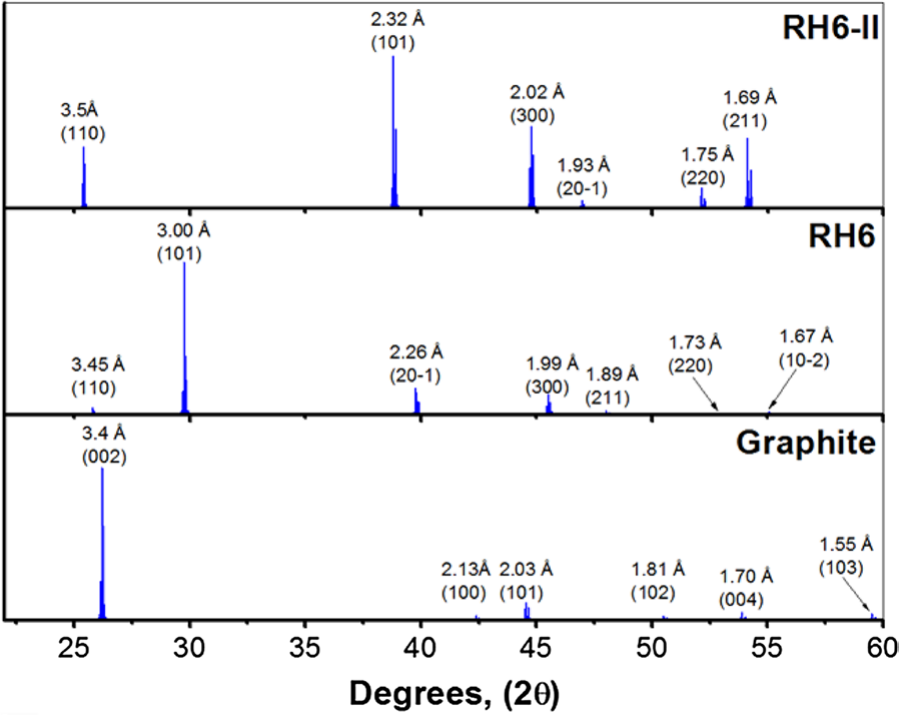
ab initio simulated XRD for the graphite, Rh6, and Rh6-II structures. The *d-spacing* along with the respective miller indices for each reflection are included in the figures

The XRD simulations carried in this work were made with the intention of identifying the exact planes having constructive interference as per the Bragg’s Law (Fig. 4). The crystal structure corresponds to trigonal cell with group number 166 and group name R3M. The structure cell parameters for the Rh6 phase are as follows: a = 6.902 Å, b = 6.902 Å, c = 3.47 Å, α = 90˚, β = 90˚, γ = 120˚. For the RH6-II, the cell group is the same 166 with the same name R3M, and the respective structure cell parameters are as follows: a = 7.012 Å, b = 7.012 Å, c = 2.509 Å, α = 90˚, β = 90˚, γ = 120˚. Examples of the models for each structure are given in Fig. 2 and the simulated XRD are given in Fig. 4. One of the mayor findings among the Rh6, Rh6-II structures, and the graphite is the first reflection that appears at similar locations; however, there are differences that can be better identified using HRTEM with resolutions as the TEAM 05. The results of the simulations are in agreement with the behavior observed in Fig. 2a by the reflection at 0.338 nm. This reflection decreases in intensity and after 2 h of milling until the constructive interference of this reflection extinguishes. After that milling time, a new wide reflection is observed at approximately 0.356 nm. This new reflection is attributed to a mix of the (101) and the (110) planes for the Rh6 and Rh6-II structures, respectively. It is possible that this reflection includes the remains of the graphitic structure.

The DFT calculations were performed on the ab initio plane-wave pseudopotential code CASTEP [48]. The GGA was used to account for exchange and correlation in the Perdew–Burke–Ernzerhof (PBE) form [49]. Both atom positions and the unit cell were relaxed to minimize the atomic forces and the total energy, such that forces were converged to 0.001 eV/A and stress residual to 0.002 GPa. A plane-wave cutoff of 310 eV was used, and Brillouin-Zone integrals were performed using a 4 × 4 × 10 Monkhort-Pack grid [50]. The Raman spectra for all the carbon polymorphs were obtained from CASTEP package using norm-conserving pseudopotentials (energy cutoff 310 eV) and the PBE-GGA exchange correlation functional.

**Fig. 5:**
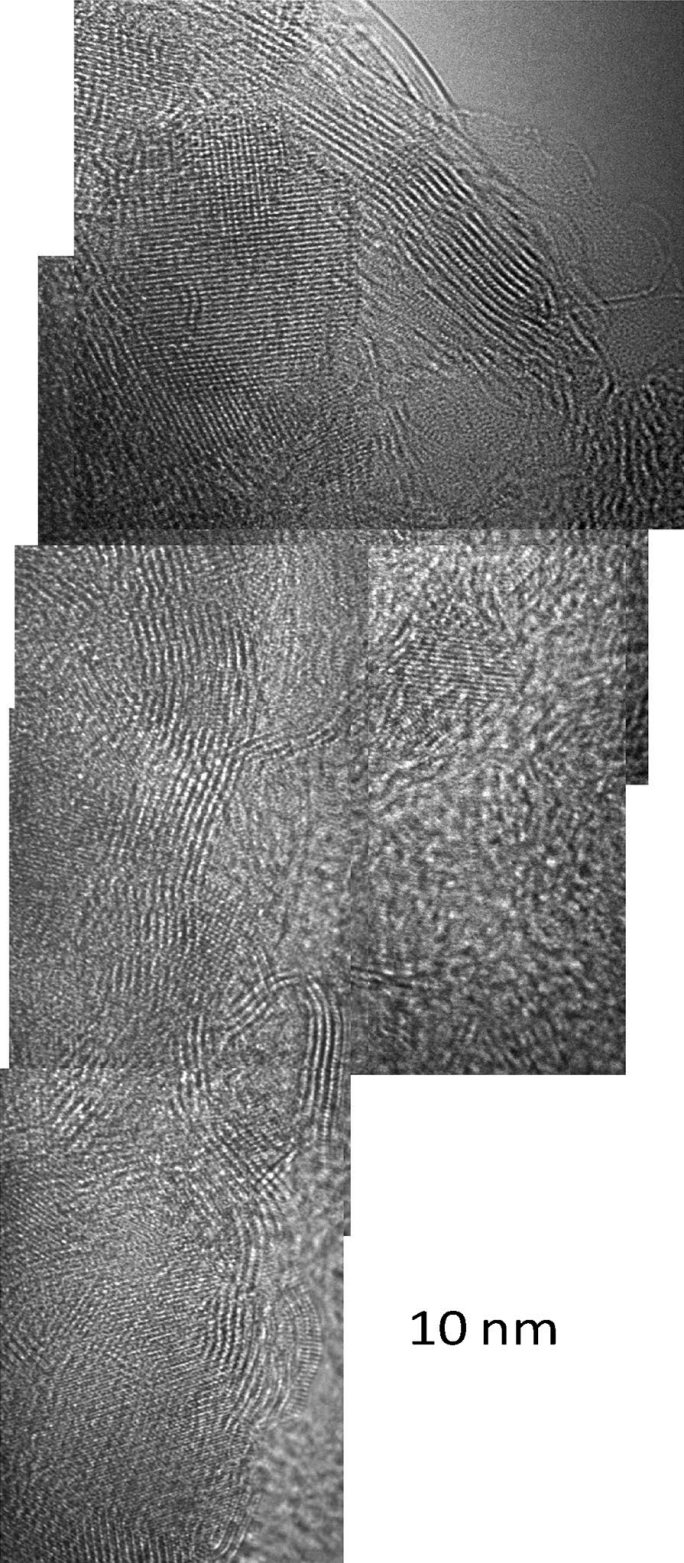
Collage showing a large area with a distribution of morphed graphenes (Rh6 and Rh6-II) particles. The area identified with the *dotted rectangle* indicated the approximate region where the analysis in Fig. 6 was performed

**Fig. 6:**
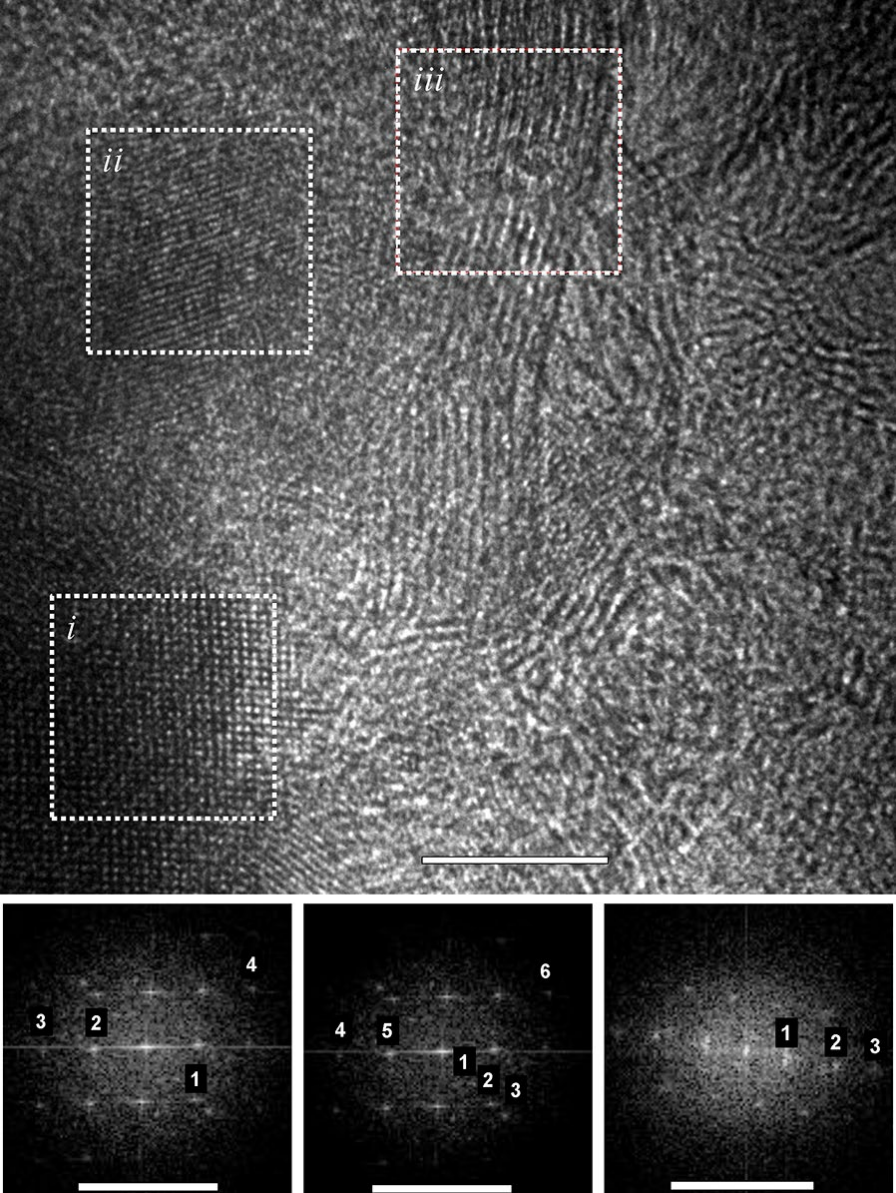
Magnified section of Fig. 5 (collage of a large area). Here, domains of graphene and morphed graphenes that were analyzed independently for the regions (*i, ii, and iii*) are shown. The analyses of (*i*), (*ii*), and (*iii*) correspond with Rh6, Rh6-II, and graphene, respectively, as shown in Table 1

Longer milling times (>2 h) increase the number of layers of graphene transforming the graphene into graphitic carbon. However, at that point the identification with Raman and XRD is no longer simple. The collage presented in Fig. 5 has as a main intention to show a large area where we can demonstrate the abundance of the mentioned phases such as graphene and more importantly morphed graphenes. This figure helps in two ways: in the first one, it offers a large area with clearly defined particles. The graphenes are layered structures, while the morphed graphenes are rather crystalline. The second important aspect of this image is to show the large abundance of the morphed graphenes. For further evidence of the differences among those particles, we strategically selected an area where we have both morphed graphene and graphene particles and it is further analyzed and presented in Fig. 6.

Three different areas/particles have been analyzed in Fig. 6. They are selected on the basis of their crystalline appearance. They are abundant in the as-milled products which is evident in the added image (Fig. 6a). The area observed in Fig. 6b has three domains which are analyzed separately using a Fast Fourier Transformation (FFT) analysis in the Digital Micrograph^®^ software. The FFT-SAEDPs are used to confirm the *d-spacing* with a higher statistics than direct measurements on specific areas along the particles. In other words, measuring the *d-spacing* in the FFT-SAEDP is more accurate and recommended. Each of the small roman numbers corresponds to a particle that is identified in Table 1. The particles identified as (*i*), (*ii*) belong to phase RH6, while area (*iii*) to Rh6-II. This identification is conducted based on our results and the *d-spacing* that is compared to the ab initio simulations for the XRD spectra.

The reflections from the FFT are matched against those *d*-*spacing* and miller indices as per the ab initio simulations. The experimental results are compared in Table 1. In Table 1, , the first two (*i* and *ii*) of the three selected particles belong to the Rh6 phase and the third one belongs to Rh6-II. The respective miller indices are provided within the table along with their respective *d*-*spacing*. It is important to point out that the reported *d*-*spacing* was measured directly over the FFT diffraction pattern to improve accuracy and the values were further confirmed measuring directly on the HRTEM images. The results can be compared to those in Fig. 4. Additionally, the identified reflections in (*iii*) are secondary reflections, it means they are repeated planes diffracting at the respective locations. This information is used here as example to show the uniqueness of each of the particles. In addition, this allows us to demonstrate that the non-graphitic structures (seen as crystalline regions in Fig. 6) are not graphite or graphitic carbon and each one is different in nature. Furthermore, the presence of the crystalline areas is not limited to the spot analyzed in Fig. 6a. Instead, we show in Fig. 5 a large number of locations with the crystalline areas.

**Fig. 7:**
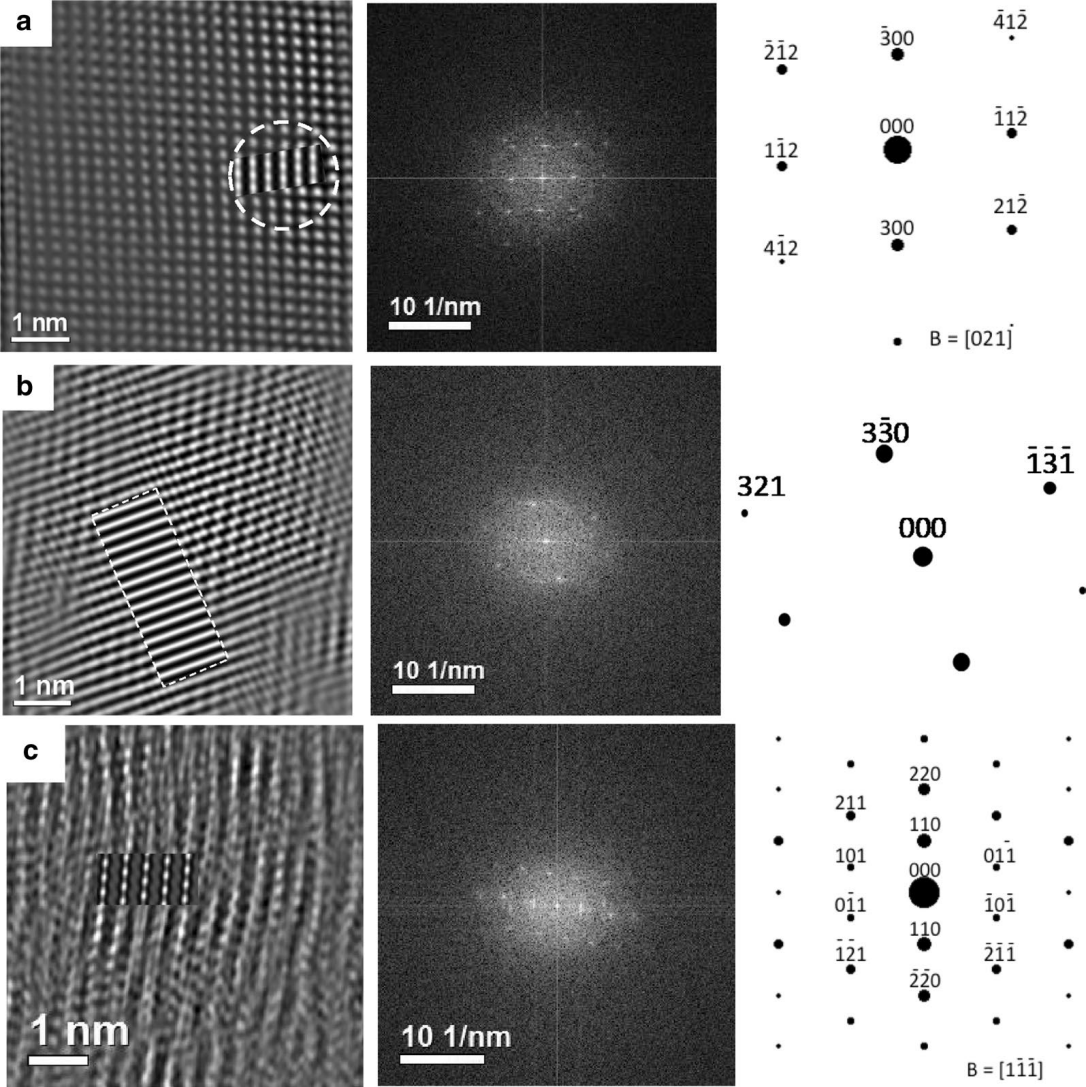
The (**a**, **b**, **c**) HRTEM images presented herein correspond to the (*i*, *ii*, and *iii*) locations in Fig. 6. The second column is the respective FFT-SEADP and the third one is the indexed pattern with its zone axis. The images are for the morphed graphenes, **a** Rh6, **b** Rh6-II, and **c** graphene. The insets in the HRTEM images are ab initio simulations

Figure 7 is used to confirm further the above-mentioned points along with the proper identification of the morphed graphenes. In this figure, we show the images for the different selected regions (*i*, *ii,* and *iii*) from Fig. 6 with their corresponding FFTs and their respective zone axis indexed diffraction pattern. Image and diffraction pattern simulations are based on data provided by Wang et al. [27]. MacTempassX and CrystalKit X are used to simulate images in the TEAM 05 microscope for the corresponding experimental conditions as well as to determine the expected diffraction patterns and atom configurations for selected orientations, respectively. The following are the TEAM 05 experimental conditions and used for simulation 80 keV, Cs = −0.015 mm, focus spread 10 Å, divergence 0.10 mRad, Cs5 = 5.5, lens aperture 1.251/Å. The resulting simulations are presented in Figs. 7 and 9.

Figure 7 shows the experimental images of each involved area together with an inset showing the image simulation for a defocus setting near 0 nm. The corresponding FFTs are also shown, they have been used to determine the corresponding zone axis and for phase identification. The small insets in each experimental image clearly match the image features for the selected defocus setting. This is possible only for the selected phases and the respective crystalline structure and zone axis, allowing phase identification in these particular areas. In this manner, area (*i*) is identified as a result of observation along a zone axis near [021] as the RH6 phase. Results for area (*ii*) are given in Fig. 7b; here the nearest zone axis is along [−1−14]. This is a zone axis rather close to a high symmetry axis in the structure of Rh6 but slightly tilted away. The magnification of the image still allows observing a network of columns in two directions but with rather small spacings between them. The image simulation in this case has been done for the precise [−1−14] orientation and only straight bars are obtained.

Figure 7c shows the results for area (*iii*). In this case, the zone axis is determined to be [1] for the phase RH6-II. The image simulation is overlapped on the filtered experimental area (*iii*) image to improve the quality and ease the comparison. Filtering the IFFT-HRTEM image helped demonstrating the almost perfect match between experimental and simulated results. Filtering only involves removing low frequencies in the FFT.

**Fig. 8:**
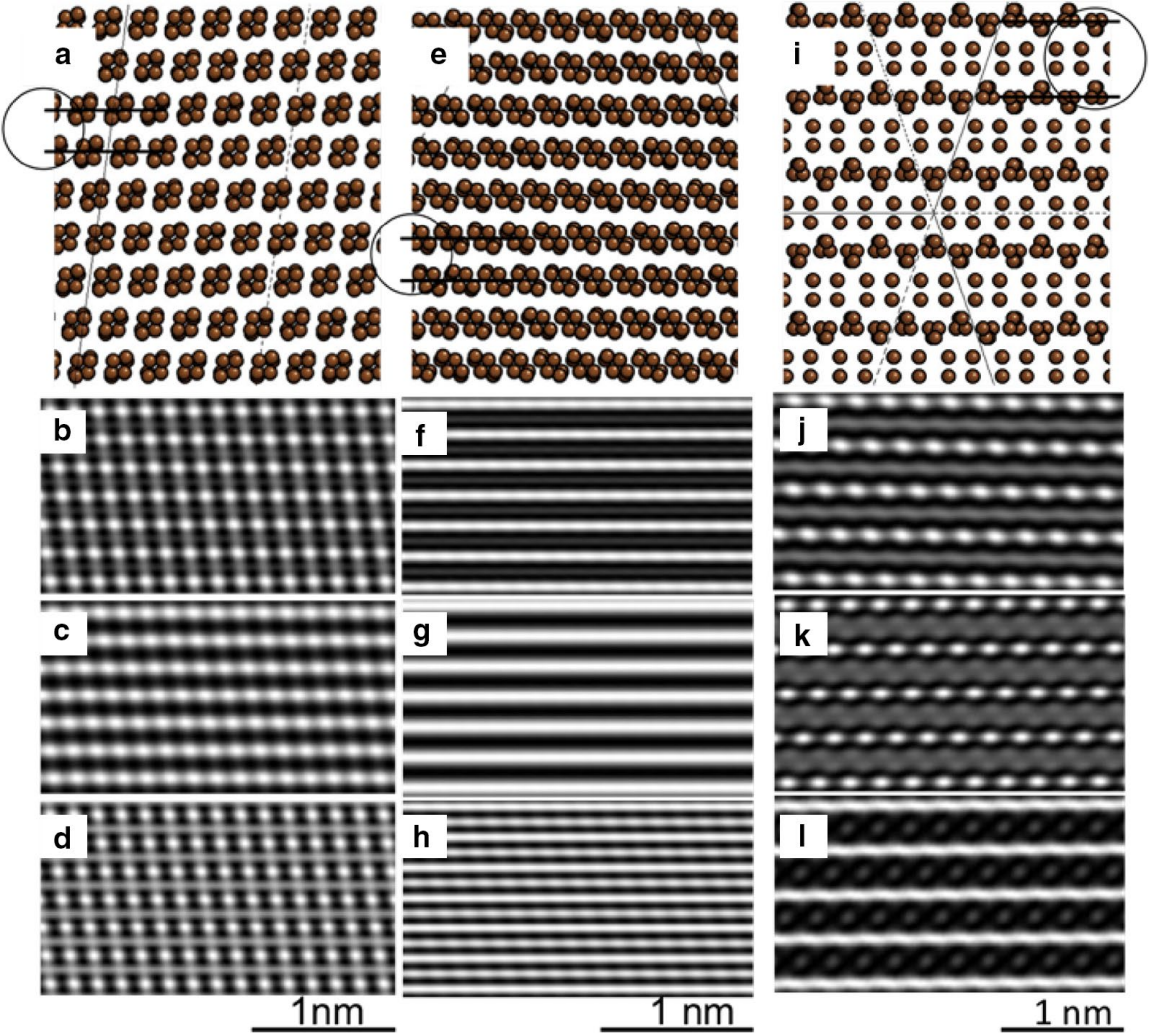
Atom arrangement and simulated images for the corresponding areas (*i*, *ii*, and *iii*) in Fig. 6. The images (**a**–**d**) correspond to area (*i*), (**e**–**h**) for area (*ii*), and (**i**–**l**) for area (*iii*). The images (**a**, **e**. and **i**) show a circle with two lines highlighting the *d*-*spacing* that are in agreement with experimental images and XRD results. The following are the respective values along with the zone axis for each area **a** 0.24 nm, B = [021]; **e** 0.21, B = [−1−14] nm and (i) 0.33 nm, B = [1−1−1]

The simulations presented in the insets of Fig. 7 are the results of the atomic arrangements expected for the two phases, Rh6 and Rh6-II. They are shown in Fig. 8 for the different zone axes involved. Figure 8a, e and i shows the atomic arrangements for the three different areas under analysis, they correspond to the phases Rh6 (Fig. 8a, e) and Rh6 II (Fig. 8i). Three different characteristic interplanar spacings are indicated by parallel lines, i.e., 0.24 nm (Fig. 8a), 0.21 nm, (Fig. 8e), and 0. 33 nm (Fig. 8i). Furthermore, three different simulated images are given in Fig. 8 for each of the involved selected areas as a function of defocus conditions. Figure 8b–d shows the results for the zone axis [021], and Fig. 8f–h for the orientation [−1−14] of the phase RH6 in areas (i) and (ii) of Fig. 6. Correspondingly, Fig. 8j–l shows the simulated images for the zone axis [1] of phase RH6-ii. The defocus conditions in all cases are −30, 20, and 70 nm starting from the top image for each series. Nevertheless, the critical part with no doubt is that the simulated images match well to the actual experimental images as presented in the superimposed insets in the HTEM, shown in Fig. 7. The identified match in the experimental and simulated images allow us to conclude the existence of the morphed graphenes and the identification of two different phases (Rh6 and Rh6-II) in the investigated samples.

The existence of the morphed structure Rh6-II is quite novel due to its bonding rotation from sp^2^ to sp^3^, which is further confirmed with XPS. The percentage of sp^2^ decreases from 95 to 81 wt% for the raw and milled products, respectively. At the same time, the presence of sp^3^ increases from 4.2 wt% sp3 to 18.2 at% sp3 for the raw and milled soot for 20 h. After that the abundance drops to 8.7 wt% sp3 in the sample milled for 50 h. The XPS results detect up to 0.7 at% Fe for samples milled up to 20 h, which is a contamination caused by wearing of the milling media. The contamination after 50 h of milling increased to approximately 3 wt%. What we can learn from the XPS results is that the presence of sp^3^ bonds increases with milling times, which we attributed it to an increase in Rh6-II.

**Fig. 9:**
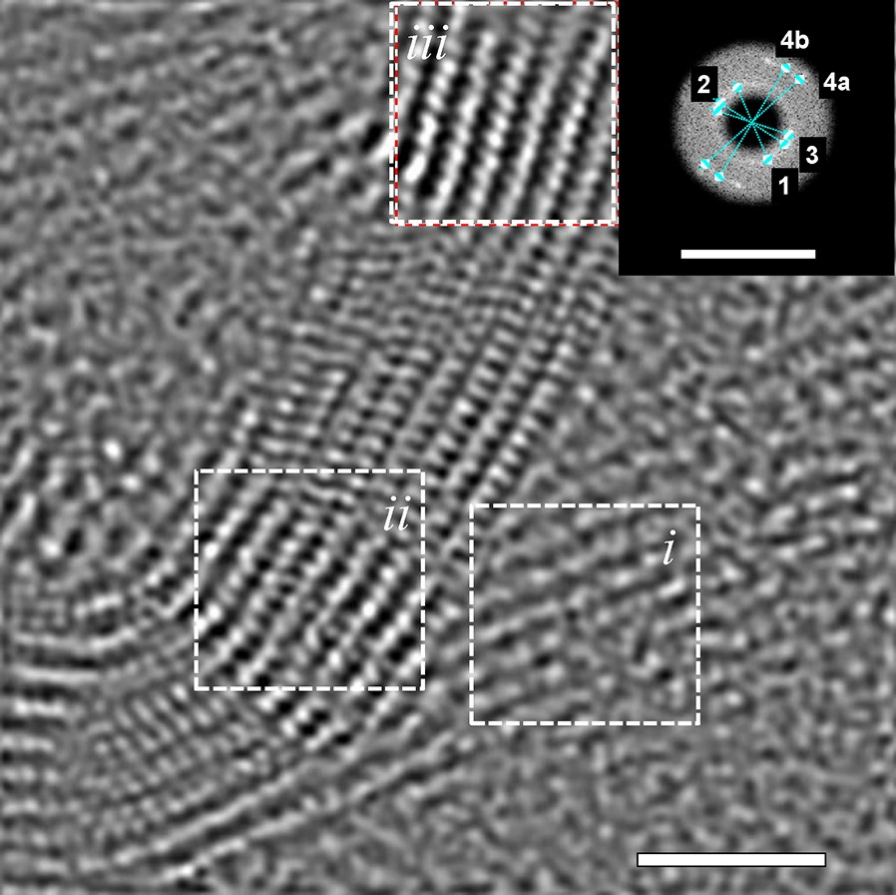
Mixture of graphitic carbon, Rh6, and Rh6-II structures observed under low-dose HRTEM. The characteristic reflections identified in the inset correspond to graphene with *d-spacings*; the respective structures in (*i*), (*ii*), and (*iii*) are graphitic carbon, Rh6, and Rh6-II. The inset is the FFT of the entire image

In Fig. 9 is presented another image where combination of phases is observed. This is important evidence because we can observe two particles developing from the same one. We believe that highly deformed graphenes developing for transform into the morphed graphenes starting by the corrugated layers followed by the switch from the sp2 to the sp3 bonding. Figure 9 shows the result of a procedure of exit wave reconstruction (using 40 images at different defoci and the software package Mac Tempas^®^), i.e., it is a phase image that gives atomic species and lattice spacings with improved resolution as compared to normal interference direct experimental images.

This is important evidence because we can appreciate a particle that is breaking into two new ones. We believe that highly deformed graphenes are the initial state to develop morphed graphenes. In Fig. 9 are identified three regions as (*i*), (*ii*), and (*iii*) with their respective FFTs. In the three regions, we measured the *d-spacing* directly over the image and we identify the following values: (*i*) 0.359, (*ii*) 0.35, and (*iii*) 0.337 nm. The *d-spacing* can also be determined using the FFT (inset) for the reciprocal distances (1), (2), and (3) with the following results: 0.363, 0.354, and 0.332 nm. From both measurements, we can conclude that the values from each approach are close confirming their differences. Using the values reported in Fig. 4, we conclude that the d-spacings are for the planes (110), (101), and (002) for the corresponding Rh6-II, Rh6, and graphitic structures. The *d*-*spacings* in 4a and 4b are 0.20 and 0.218 nm, which are the perpendicular distance. Based on this evidence, we conclude that the plastic deformation during milling sponsors the transformation of graphene into Rh6 and then at higher levels of deformation Rh6 may transform into the Rh6-II structure. Nevertheless, the development of a phase such as Rh6 ii does not have to depend on a sequence but rather on the bonding and thus on the very different possibilities that a highly aleatory processing such as mechanical milling can offer.

The same *d*-*spacings* and differences were measured with conventional HRTEM images and XRD, con1cluding that in order to achieve the right accuracy to interpret the results, we needed an instrument with higher resolution. Here is where the resolution on the TEAM 05 is key because it is clearly evident that the fine differences among the results presented could not be resolved with conventional instruments.

## Conclusions

We have presented the HRTEM evidence under low dose for conventional structures of carbon (graphene and graphitic carbon) as well as the newly identified carbon nanostructures known as morphed graphenes. The use of atomic resolution TEM is of paramount interest because it is the only characterization tool available to distinguish the differences among Rh6 and Rh6-II. The nanostructures have been predicted previously and some features are identified using Raman, XRD, and XPS, but neither of those methods has been capable of proving the existence of the aforementioned phases. Here a complementary set of experimental and numerical tools is used to clearly demonstrate the presence of the two morphed graphene phases and their uniqueness when compare to graphene or other carbon allotropes. Furthermore, electron microscopy investigations in low-dose conditions are key to allow a thorough characterization of the morphed graphenes, while preserving them in damage-free condition (intact).
